# A fast BK-type K_Ca_ current acts as a postsynaptic modulator of temporal selectivity for communication signals

**DOI:** 10.3389/fncel.2014.00286

**Published:** 2014-09-17

**Authors:** Tsunehiko Kohashi, Bruce A. Carlson

**Affiliations:** ^1^Department of Biology, Washington University in St. LouisSt. Louis, MO, USA; ^2^Division of Biological Science, Graduate School of Science, Nagoya UniversityNagoya, Japan

**Keywords:** electrosensory, temporal processing, BK channel, calcium-activated pottasium channel, spike frequency adaptation, synaptic integration

## Abstract

Temporal patterns of spiking often convey behaviorally relevant information. Various synaptic mechanisms and intrinsic membrane properties can influence neuronal selectivity to temporal patterns of input. However, little is known about how synaptic mechanisms and intrinsic properties together determine the temporal selectivity of neuronal output. We tackled this question by recording from midbrain electrosensory neurons in mormyrid fish, in which the processing of temporal intervals between communication signals can be studied in a reduced *in vitro* preparation. Mormyrids communicate by varying interpulse intervals (IPIs) between electric pulses. Within the midbrain posterior exterolateral nucleus (ELp), the temporal patterns of afferent spike trains are filtered to establish single-neuron IPI tuning. We performed whole-cell recording from ELp neurons in a whole-brain preparation and examined the relationship between intrinsic excitability and IPI tuning. We found that spike frequency adaptation of ELp neurons was highly variable. Postsynaptic potentials (PSPs) of strongly adapting (phasic) neurons were more sharply tuned to IPIs than weakly adapting (tonic) neurons. Further, the synaptic filtering of IPIs by tonic neurons was more faithfully converted into variation in spiking output, particularly at short IPIs. Pharmacological manipulation under current- and voltage-clamp revealed that tonic firing is mediated by a fast, large-conductance Ca^2+^-activated K^+^ (K_Ca_) current (BK) that speeds up action potential repolarization. These results suggest that BK currents can shape the temporal filtering of sensory inputs by modifying both synaptic responses and PSP-to-spike conversion. Slow SK-type K_Ca_ currents have previously been implicated in temporal processing. Thus, both fast and slow K_Ca_ currents can fine-tune temporal selectivity.

## INTRODUCTION

Processing temporal patterns of stimulus-dependent neural activity is fundamental for sensory perception (Carr, 1993; Cariani, 2001; Lestienne, 2001; Panzeri et al., 2010). This processing has been well studied in sensory pathways where neurons exhibit selective responses to the temporal structure of sensory inputs (Grothe, 1994; Rose and Fortune, 1999; Edwards et al., 2002; Edwards and Rose, 2003; Pluta and Kawasaki, 2010; Huetz et al., 2011; Baker et al., 2013). A variety of synaptic mechanisms are suggested to establish this selectivity (Grothe, 1994; Buonomano, 2000; Fortune and Rose, 2001; Zucker and Regehr, 2002; Klyachko and Stevens, 2006; Edwards et al., 2007, 2008; George et al., 2011; Rose et al., 2011). Intrinsic properties of postsynaptic membranes, such as voltage-gated and Ca^2+^-activated currents, can also contribute to temporal filtering of synaptic inputs (Fortune and Rose, 1997; Trussell, 1999; Hutcheon and Yarom, 2000; Fortune and Rose, 2003; Carlson and Kawasaki, 2006; Ellis et al., 2007; Mehaffey et al., 2008; Ponnath and Farris, 2010; Ashida and Carr, 2011; O’Donnell and Nolan, 2011; Golding, 2012). However, less is known about how the combination of intrinsic properties and synaptic filtering together generate selective spiking responses to temporal patterns of synaptic input.

Here, we addressed this question by studying the electrosensory pathway of mormyrid electric fishes. A striking advantage of this pathway is that behaviorally relevant temporal patterns of network activity can be reproduced in reduced *in vitro* preparations, in which network mechanisms are readily accessible at a subcellular level (George et al., 2011; Ma et al., 2013). Mormyrids generate an all-or-none electric organ discharge (EOD) and communicate with other fish by varying interpulse intervals (IPIs) between EODs (Carlson, 2006). These signals are analyzed by a sensory pathway dedicated to electric communication (Xu-Friedman and Hopkins, 1999; Baker et al., 2013). The timing of each EOD from a neighboring fish is precisely encoded into the spike times of peripheral electroreceptors (Hopkins and Bass, 1981; Lyons-Warren et al., 2012), thereby encoding IPIs into interspike intervals. This information is relayed to the midbrain posterior exterolateral nucleus (ELp), where afferent spike trains are filtered to establish single-neuron IPI tuning (Carlson, 2009). Behaviorally relevant synaptic input patterns to ELp can be generated by directly stimulating the adjacent anterior exterorateral nucleus (ELa; Carlson, 2009; George et al., 2011).

Previously, we capitalized on this advantage to study synaptic mechanisms and network interactions that establish IPI tuning using *in vitro* preparations containing ELp and ELa (George et al., 2011; Ma et al., 2013). However, the intrinsic properties of ELp neurons have not previously been studied. Using a whole-brain preparation in which local ELp circuitry remains intact (Ma et al., 2013), we examined contributions of intrinsic excitability to shaping synaptic potentials and converting them into action potentials to drive local computations for decoding IPIs. Our results suggest that a fast, large-conductance calcium-activated K^+^ current controls spike frequency adaptation as well as subthreshold postsynaptic potentials (PSPs) in ELp neurons, resulting in significant postsynaptic modulation of IPI tuning.

## MATERIALS AND METHODS

### ANIMALS

We used individuals of the weakly electric mormyrid fish *Brienomyrus brachyistius* of both sexes, ranging from 6.1 to 11.5 cm in fork length. The fish were obtained through commercial venders and housed in community tanks with a 12 h:12 h light/dark cycle, temperature of 25–28°C, pH of 6–7, and water conductivity of 200–400 μS/cm. Fish were fed live black worms four times per week. All procedures were in accordance with guidelines established by the National Institutes of Health and were approved by the Animal Care and Use Committees at Washington University in St. Louis.

### WHOLE-CELL RECORDING FROM ELp NEURONS

In most of the experiments, we used an *in vitro* whole-brain preparation developed in a previous study (Ma et al., 2013). In brief, we anesthetized fish in 300 mg/L MS-222, and then performed a craniotomy in ice-cold, oxygenated artificial cerebrospinal fluid (ACSF; composition in mM: 124 NaCl, 2.0 KCl, 1.25 KH_2_PO_4_, 24 NaHCO_3_, 2.6 CaCl_2_, 1.6 MgSO_4_.7H_2_O, and 20 glucose, pH 7.45; osmolarity: 310 mOsm) containing 1 mM kynurenic acid (KA) to reduce potential excitotoxicity. The valvula cerebellum and dorsal part of the hindbrain were removed by suction while in ACSF, leaving the remainder of the brain intact. The brain was then removed and was allowed to equilibrate in oxygenated ACSF containing 0.5 mM KA at room temperature (23–27°C). One hour later, the brain was transferred to a recording chamber (RC-26GPL; Warner Instruments, Hamden, CT, USA) containing the equilibrating solution and secured by two slice anchors (Warner Instruments SHD-26GH) placed on the bottom and the top of the brain. We used cured silicone on the top of the recording chamber to hold the top anchor in place. The chamber was then placed on a recording platform (Burleigh Gibraltar; EXFO, Mississauga, ON, Canada). On the platform, the brain was continuously perfused (flow rate: approximately 1 ml/min) with oxygenated ASCF at room temperature. One additional hour of equilibration was allowed for KA to wash out before we started recording. In some experiments, we added tetrodotoxin (TTX) citrate (1 μM), NiCl_2_ (1–3 mM), and/or 4-aminopyridine (4-AP; 1 mM) to the perfusate, followed by washout in ACSF. For these fast-acting drugs, the effects of wash-in or washout were measured after two minutes of perfusion.

In experiments using a specific large-conductance calcium-activated K^+^ channel (BK) blocker, paxilline (10 μM in 0.04% DMSO in ACSF), we used an *in vitro* slice preparation (George et al., 2011) to facilitate penetration of this slow-acting drug into the tissue. In brief, we cut horizontal sections (300 μm) in ice-cold ACSF containing 1 mM KA using an oscillating tissue slicer (VF-200 Compresstome; Precisionary Instruments, San Jose, CA, USA). Before we started recording, the sections were allowed to equilibrate as with the whole-brain preparation. Care was taken to ensure that we recorded from the same area of ELp as in the experiments using the whole-brain preparation.

We visualized ELp neurons using transmitted light microscopy in an upright microscope (BX51WI; Olympus, Tokyo, Japan) in combination with a Newvicon tube camera (NC-70; DAGE-MTI, Michigan City, IN, USA). We performed whole-cell intracellular recordings using filamented, borosilicate patch pipettes (1.00 mm outer diameter; 0.58 mm inner diameter) with tip resistances of 4–8 MΩ. The electrode internal solution contained the following (in mM): 130 K gluconate, 5 EGTA, 10 HEPES, 3 KCl, 2 MgCl_2_, 4 Na_2_ATP, 5 Na_2_ phosphocreatine, and 0.4 Na_2_GTP, pH 7.3–7.4 (osmolarity: 280–290 mOsm). Recordings were amplified using a MultiClamp 700B amplifier (Molecular Devices, Union City, CA, USA), digitized at a sampling rate of 50 kHz (Molecular Devices Digidata 1440A) and saved to disk (Molecular Devices Clampex v10.2). TTX citrate and paxilline were obtained from Tocris (Bristol, UK) and Cayman (Ann Arbor, MI, USA), respectively, and all other chemicals were purchased from Sigma (St. Louis, MO, USA).

### CURRENT-INJECTION AND SPIKE ANALYSIS

We only used data from neurons that had a resting potential more hyperpolarized than –50 mV. To determine resting passive membrane properties, we injected a small, 600 ms hyperpolarizing square current pulse (–3 to –20 pA; resulted in less than 5 mV voltage shift). We calculated the membrane time constant, input resistance, and membrane capacitance from the time constant and amplitude of a single-exponential fit. We evoked action potentials by applying stepwise depolarizing current injections. We defined threshold current as the minimum current amplitude evoking an action potential, determined with a resolution of at least 0.1× threshold. We classified ELp neurons into two types, according to firing patterns in response to depolarizing current injections with a duration of 600 ms and a current amplitude of 3× threshold: we defined tonic neurons as those which fired throughout the entire duration of the current pulse, and phasic neurons as those which stopped firing in the middle of the current step. We analyzed the waveforms of action potentials elicited at the threshold current intensity. The parameters used to characterize action potential waveforms were: threshold voltage [*V*_T_; the voltage at which d*V*/d*t* reached 5% of the maximum (Jackson et al., 2004; Khaliq and Raman, 2006)], amplitude (measured from *V*_T_ to the peak), spike latency (time elapsed from the onset of current injection to the peak of the action potential), rise- and fall-time (time elapsed from the upward *V*_T_ crossing to the peak, and from the peak to the downward *V*_T_ crossing, respectively), and half-width (duration measured at 50% of the spike amplitude). We also measured the height and time to the trough of the afterhyperpolarization (AHP; amplitude and onset, respectively, measured from the downward *V*_T_ crossing to the AHP minimum). The AHP was measured from the first action potential elicited by the lowest current intensity that evoked multiple action potentials so that we could define the AHP minimum as the minimum voltage between action potentials. We chose this method to measure AHP amplitude because the behavior of membrane potentials/currents that directly lead the following spikes is relevant to when neurons actually perform temporal processing.

### IPI TUNING

Interpulse interval (IPI) tuning curves of synaptic responses were constructed as previously described (Carlson, 2009; George et al., 2011; Ma et al., 2013). To stimulate excitatory inputs to ELp, we placed an array of stimulus electrodes in ELa, just anterior to the ELp border (George et al., 2011; Ma et al., 2013). The array consisted of four channels of bipolar stimulation (eight electrodes total) in the form of either a cluster electrode or matrix electrode (models CB and MX, respectively, FHC, Bowdoin, ME, USA). We delivered isolated, biphasic square current pulses (100 μs total duration; less than 200 μA amplitude) through four separate isolated pulse generators (model 2100; A-M Systems, Sequim, WA, USA). We stimulated ELa with single pulses as well as stimulus trains of 10 pulses with constant IPIs ranging from 10 to 100 ms. If there were no spikes in response to synaptic stimulation, we averaged the response traces across stimulus repetitions to obtain a single averaged response trace. Next, we averaged the maximum depolarizations in response to the 2nd through 10th pulses to obtain a single average maximum depolarization for each IPI. Finally, the resulting maximum depolarizations for all 10 IPIs were normalized to the largest average response measured. If there was any spiking in response to synaptic stimulation, we applied a median filter (width: 1.5 ms) to each trace to remove spikes (Jagadeesh et al., 1993; Carlson, 2009; George et al., 2011; Ma et al., 2013). When the spike-filtered traces were averaged in the same way as the spike-free traces, peak synaptic depolarization and sharp AHP in different traces sometimes canceled each other because of temporal jitter in spike times. To avoid this effect, we first collected the maximum depolarizations in response to the 2nd through 10th pulse for each trace and then averaged them across traces to obtain the average maximum depolarization for each IPI. We also constructed tuning curves based on spiking responses. We averaged the number of action potentials in response to the 2nd through 10th stimulus pulses for each IPI, and normalized it to the largest average response across all 10 IPIs (Carlson, 2009).

We categorized the tuning of neurons by identifying the IPIs that elicited responses ≥85% of the maximum response (Carlson, 2009; George et al., 2011; Ma et al., 2013). Using a linear extrapolation between adjacent points, we identified each IPI at which a tuning curve crossed the 85% criterion. We classified neurons as “all-pass” if the responses to all IPIs were≥85% of maximum. If the tuning curve had a single point that crossed the 85% criterion, we classified the neuron as “low-pass” or “high-pass” depending on whether long or short IPIs elicited responses ≥85%, respectively. If the tuning curve had two points that crossed the 85% criterion, we classified the neuron as “band-pass” or “band-stop” according to whether intermediate IPIs resulted in responses above or below 85%, respectively. We classified the neuron as “complex” if the tuning curve had three or more points that crossed the 85% criterion. To investigate subtle differences among tuning curves within the same category, tuning curves were tested for the following quantitative criteria before inclusion in statistical analyses. Synaptic responses to the first stimulus in each stimulus train was used to evaluate the stability of stimulation and recording while varying IPIs. We calculated the coefficient of variation of the first response amplitude across IPIs and included only tuning curves having a coefficient less than 0.3 in subsequent analyses. Also, we included only tuning curves that showed an average maximum depolarization of more than 3 mV at the best IPI because this study focused on contributions of depolarization-gated currents.

Some neurons were tested more than twice for assessing IPI tuning by changing the combination of stimulus channels used for ELa stimulation and/or the amplitude of stimulus current pulses. If single stimulus pulses at different stimulus settings evoked synaptic responses in the same cell that differed by more than 30% in peak latency or more than 30% in peak amplitude, we assumed that the stimulus settings activated different local circuits and included both tuning curves in analyses.

### VOLTAGE CLAMP RECORDING

Some neurons were tested for voltage-dependent currents in voltage-clamp mode after being tested for firing pattern in current-clamp mode. The membrane potential was held at –65 mV, close to the resting potential, between each test. To maximally release inactivation of voltage-gated channels, the holding potential was hyperpolarized to –85 mV 200 ms before applying test potentials of 100 ms duration (ranging from –95 mV to +35 mV in 10 mV increments). The multipolar morphology of ELp neurons (Xu-Friedman and Hopkins, 1999; George et al., 2011; Ma et al., 2013) and deep ACSF level due to the thick tissue of the whole-brain preparation made capacitance compensation difficult. This issue resulted in poorly compensated capacitive currents at the beginning of voltage steps (typically within 1 ms of voltage-step onset), which masks fast whole-cell currents. Therefore, to remove the capacitive current and leak current, and extract voltage-gated currents, we considered current responses between –95 and –75 mV as passive, estimated the conductance of the response by linear regression, and subtracted the conductance from the rest of the original current traces at all test potentials. The validity of this procedure for removing leak and capacitive currents was confirmed by a linear current–voltage relationship below –65 mV in every cell we tested (measured during TTX application). In particular, the fast transient currents revealed by small hyper- or depolarization steps changed linearly with voltage (*r*^2^ = 0.99–1.0, at 0.5 ms after voltage step onset). This observation indicates that these fast transient currents were indeed capacitive currents, which we could remove through subtraction.

We also found that slower components exhibited a linear current–voltage relationship below –65 mV in every cell we tested (*r*^2^ = 0.94–1.0, at 2 and 15 ms after step onset). This suggests that leak conductance was constant between –95 and –65 mV. However, the leak conductance could be different during more depolarized voltage steps if ELp neurons express channels activated at hyperpolarized potentials, such as hyperpolarization activated cation (*I*_h_) channels and inwardly rectifying potassium (K_ir_) channels. *I*_h_ currents are slow: to our knowledge, activation/deactivation time constants of *I*_h_ currents shorter than 25 ms have never been reported, and they are typically several hundred milliseconds (reviewed in Pape, 1996; Santoro and Tibbs, 1999; Wahl-Schott and Biel, 2009). Because we focused on currents at timescales much shorter than this (0.5–15 ms), HCN currents should not lead to errors in our current measurements. K_ir_ channels generate large inward conductances below the potassium equilibrium potential (*E*_k_) but still pass small outward currents above *E*_k_, also in a voltage-dependent manner (reviewed in Nichols and Lopatin, 1997; Hille, 2001; Hibino et al., 2010). Therefore, even if our voltage steps never activated large inward K_ir_ conductances (calculated *E*_k_: –95 mV based on the intracellular whole-cell solution and external ACSF), differences in the small outward K_ir_ conductance at different voltage steps could result in errors from our subtraction estimation method. Nevertheless, it is important to note that Ni^2+^-sensitive currents that were the focus of this study would not be affected by these errors because any K_ir_ and *I*_h_ conductances would be canceled by subtracting the currents obtained during combined application of TTX and Ni^2+^ (+TTX+ Ni^2+^) from those obtained during TTX-only (+TTX) application.

### STATISTICS

All statistical analyses were performed using Statistica 6.1 (StatSoft, Tulsa, OK, USA) or SigmaPlot 12 (Systat Software, San Jose, CA, USA). Logarithmic-transformation was applied when a data set failed the Shapiro–Wilk test for normality (*p* < 0.01). Values are reported as the mean ± SEM.

## RESULTS

### TONIC AND PHASIC FIRING PATTERNS OF ELp NEURONS

**FIGURE 1:**
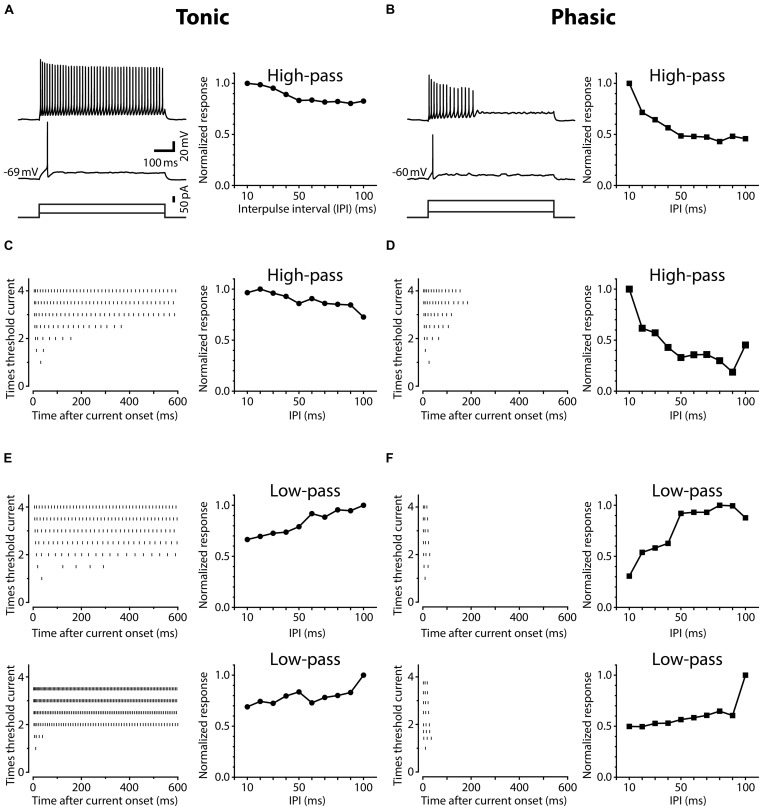
**Neurons in the posterior exterolateral nucleus (ELp) exhibit two distinct firing patterns. (A)** Left, spiking responses of a weakly adapting (tonic) ELp neuron to current steps of 600 ms duration (bottom) at threshold (middle) and three-times threshold (top) current intensities. Right, high-pass tuning curve obtained from the same neuron. The plot shows the average maximum amplitude of individual postsynaptic potentials (PSPs) in response to bipolar stimulation of the anterior exterorateral nucleus (ELa) at interpulse intervals (IPIs) ranging from 10 to 100 ms. The amplitude is further normalized by the largest maximum depolarization across IPIs. See Section “Materials and Methods” for details. Scale bars also apply to **(B)**. **(B)** Spiking response of a strongly adapting (phasic) neuron (left) that exhibited high-pass tuning to ELa stimulation (right). **(C–F)** Examples of three additional tonic **(C,E)** and three additional phasic **(D,F)** neurons that showed high-pass **(C,D)** or low-pass **(E,F)** responses. Left: Raster plots show the times of action potential peaks during current injection (abscissa) at different current amplitudes, which were normalized to the threshold intensity (ordinate).

Neurons in the ELp exhibited noticeable variation in spike frequency adaptation during prolonged depolarization. **Figure 1** shows action potential timing in ELp neurons in response to depolarizing current injection with varying intensities (left panels), accompanied with the IPI tuning of synaptic potentials of the same cells in response to bipolar stimulation of ELa (right panels). We divided ELp neurons into two classes based on the degree of spike frequency adaptation. The majority of neurons increased the number of action potentials as the depolarizing current amplitude increased and eventually spiked continuously throughout the current step (**Figures 1A,C,E**). The others showed strong spike frequency adaptation and stopped firing in the middle of the current step (typically within 300 ms after current onset; **Figures 1B,D,F**). The latter never fired continuously over the range of depolarizing currents (up to seven-times threshold) tested. We defined weakly adapting neurons that exhibited continuous spiking during a 600 ms current step at three-times threshold current intensity as tonic neurons, and strongly adapting neurons that stopped firing in the middle of this current step as phasic neurons. Out of 112 ELp neurons we recorded from in the present study, 92 (82%) were classified as tonic neurons and 20 (18%) were classified as phasic neurons.

### TONIC NEURONS CONVERT IPI TUNING OF SYNAPTIC INPUT INTO SPIKE OUTPUT MORE FAITHFULLY THAN PHASIC NEURONS

**FIGURE 2:**
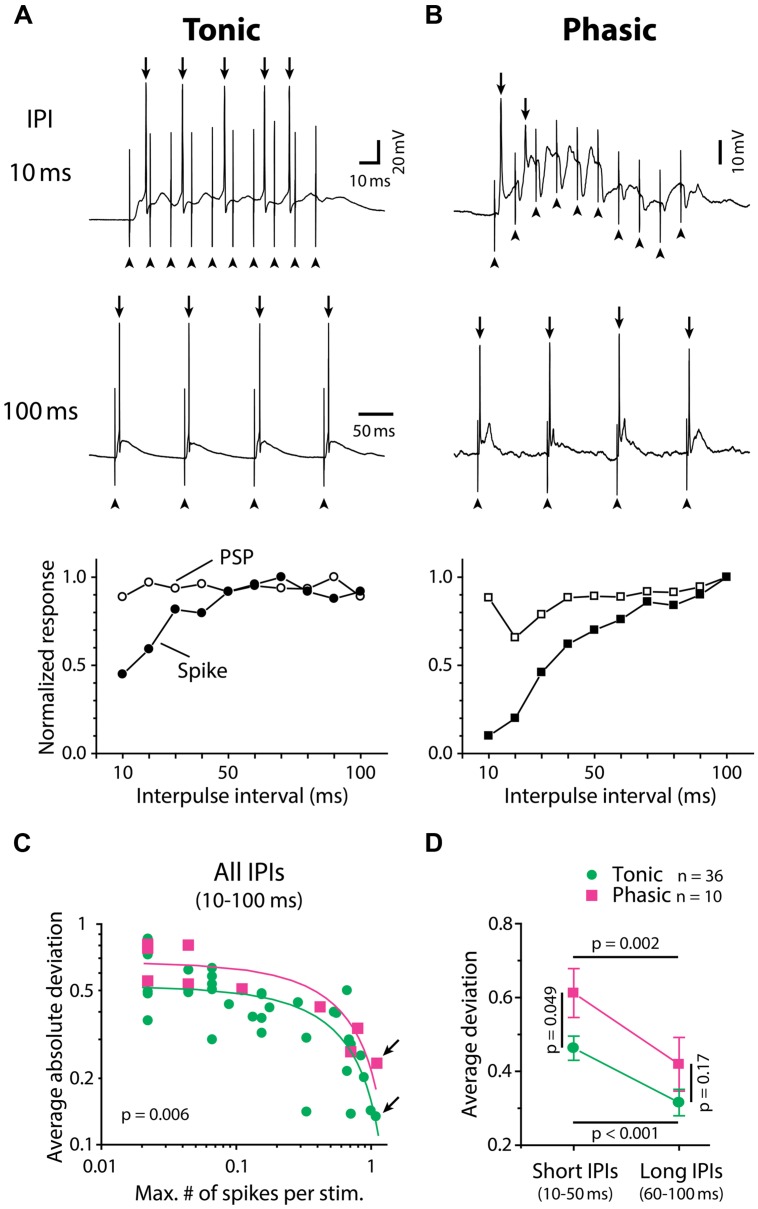
**Tonic neurons convert IPI tuning of synaptic inputs into spike output with higher fidelity than phasic neurons. (A,B)** Synaptically evoked spiking responses of a tonic neuron **(A)** and a phasic neuron **(B)** to ELa stimulation with 10 (top) and 100 (middle) ms IPIs. In these examples, both neurons fired action potentials (arrow) in response to every stimulus at 100 ms IPI. However, at 10 ms IPI, the phasic neuron rapidly adapted to stop firing after one full-size and one attenuated action potential out of ten stimuli while the tonic neuron still exhibited five full-size action potentials. Arrowhead, stimulus artifact. Horizontal scale bars refer to both **(A)** and **(B)**. Bottom, IPI tuning curves obtained from the same cells, which were plotted for average number of action potentials (filled symbols; spike) and average maximum depolarization (open symbols; PSP). Both tuning curves were normalized by dividing by the maximum response across intervals. To generate PSP tuning curves, the raw membrane potential trace containing action potentials was median-filtered to remove high-frequency components due to spikes and estimate the underlying synaptic potentials (see Materials and Methods for details). **(C,D)** Spike tuning curves of tonic neurons (green circle) are more similar to their PSP tuning curves compared to phasic neurons (magenta square). The deviation between spike and PSP tuning, defined as normalized PSP response minus normalized spike response at the same IPI, was plotted in two different ways in **(C)** and **(D)**. Legend in **(D)** also applies to **(C)**. **(C)** In both tonic and phasic neurons, the average of absolute values of the deviation across all IPIs (10–100 ms) was negatively correlated with the maximum number of spikes across IPIs (abscissa: divided by the number of stimulation, or average number of spikes at best IPI; *r* < –0.7, *p* < 0.01; green and magenta lines represent linear regression lines for tonic and phasic neurons, respectively). Regardless of how many action potentials the recorded neuron fired during the stimulus train, the average absolute deviation was consistently smaller in tonic neurons than in phasic neurons (ANCOVA; *p* = 0.006). This suggests that, during the process of converting synaptic inputs into spike output, IPI tuning curves are less distorted in tonic neurons than in phasic neurons throughout a range of synaptic activation. Arrows indicate data obtained from **(A)** and **(B)**. **(D)** Average of actual deviation, not absolute value, at short (10–50 ms) IPIs was larger than that at long IPIs (60–100 ms) in both tonic and phasic neurons. Because positive deviation reflects a spiking response that is suppressed relative to the synaptic response, this result indicates low-pass filtering of PSP tuning into spike tuning in both tonic and phasic neurons. Compared to tonic neurons, the deviation of phasic neurons was larger at short IPIs but not at long IPIs, suggesting that the low-pass filtering effect is more prominent in phasic neurons. Multiple comparison analysis was performed by the Holm–Sidak *post hoc* method following two-way repeated measures ANOVA. Black line indicates the pair of comparison and corresponding *p* value.

Tuning of ELp neurons to temporal patterns of ELa stimulation reflects tuning of ELp neurons to sensory stimulation patterns *in vivo* (Carlson, 2009; George et al., 2011; Ma et al., 2013), providing us an opportunity to investigate the relationship between IPI tuning and spike frequency adaptation of ELp neurons in behaviorally relevant ways. Phasic neurons exhibited strong spike frequency adaptation during synaptically evoked depolarizations. **Figures 2A,B** exemplify the spiking activities of a tonic and a phasic neuron, respectively, in response to ELa stimulation with 10 ms and 100 ms IPIs. At 100 ms IPIs, both neurons reliably fired action potentials in response to each stimulus pulse. However, at 10 ms IPIs, the action potentials of the phasic neuron rapidly adapted even though the membrane potential progressively depolarized. By contrast, the tonic neuron continued firing action potentials throughout the stimulus train. To quantify how IPI tuning for synaptic potential (PSP IPI tuning) is converted into IPI tuning for spike numbers (spike IPI tuning) by tonic and phasic neurons, we directly compared spike tuning curves with PSP tuning curves resulting from the same responses. We estimated synaptic potentials underlying action potentials by applying a median filter (1.5 ms width) to remove rapid voltage changes during spikes (see Materials and Methods for details). In the exemplified neurons (**Figures 2A,B**, bottom), PSP and spike tuning curves showed a greater discrepancy, particularly at shorter IPIs, in the phasic neuron compared to the tonic neuron.

To quantify the discrepancy between spike and PSP tuning curves, we calculated the difference between normalized spike and PSP responses at each IPI (deviation, defined as normalized PSP response minus normalized spike response). **Figure 2C** shows the averaged absolute value of deviations across IPIs for all neurons, plotted against the average number of spikes per stimulus pulse at the best IPI (tonic: *n* = 36, phasic: *n* = 10). The average absolute deviation was negatively correlated with spike number for both tonic (*r* = –0.72, *p* < 10^-4^) and phasic (*r* = –0.85, *p* = 0.002) neurons. Regardless of how many spikes were evoked by ELa stimulation, tonic neurons exhibited consistently lower deviations than phasic neurons [ANCOVA, *F*_(1,43)_ = 8.25, *p* = 0.006], suggesting that tonic neurons convert PSP tuning curves more faithfully into spike tuning curves throughout the behaviorally relevant range of synaptic activation.

Synaptic excitation arriving at short IPIs summates to establish a prolonged depolarization (George et al., 2011) that will readily cause spikes to adapt. On the other hand, longer IPIs allow more time for neurons to repolarize and thus recover from adaptation between each stimulus pulse. To examine the effect of the IPI-dependency of spike frequency adaptation on PSP-to-spike conversion, we compared deviations of tonic and phasic neurons at different IPIs (**Figure 2D**), this time leaving the signs of deviation values (tested with two-way repeated measures ANOVA followed by the Holm–Sidak multiple comparison analysis). Here, positive deviation represents normalized spiking responses that are suppressed relative to normalized PSP responses whereas negative deviation represents normalized spiking responses that are enhanced relative to normalized PSP responses. Both tonic and phasic neurons showed greater deviation at short IPIs (average of 10–50 ms IPIs) compared to long IPIs (average of 60–100 ms IPIs; tonic: *t* = 5.03; *p* < 0.001, phasic: *t* = 3.23; *p* = 0.002), indicating low-pass filtering of IPI tuning during the PSP-to-spike conversion. In addition, the deviation at short IPIs was higher in phasic neurons compared to tonic neurons (*t* = 2.01, *p* = 0.049), but there was no significant difference at long IPIs (*t* = 1.39, *p* > 0.1). These results suggest that phasic neurons low-pass filter PSP tuning more strongly than tonic neurons in generating spike output.

### PHASIC NEURONS EXHIBIT SHARPER IPI TUNING THAN TONIC NEURONS

We encountered all types of previously reported PSP IPI tuning patterns (Carlson, 2009; George et al., 2011; Ma et al., 2013) in this study: “all-pass” which responded equally well to all IPIs between 10 and 100 ms (tonic: *n* = 7, phasic: *n* = 0); “low-pass” which responded preferentially to long IPIs (tonic: *n* = 16, phasic: *n* = 5); “high-pass” which responded preferentially to short IPIs (tonic: *n* = 41, phasic: *n* = 6); “band-pass” which responded preferentially to intermediate IPIs (tonic: *n* = 3, phasic: *n* = 3); “band-stop” which responded preferentially to both long and short, but not intermediate IPIs (tonic: *n* = 9, phasic: *n* = 2); and “complex” which responded preferentially to multiple IPI ranges (tonic: *n* = 18, phasic *n* = 7). The relative numbers of different tuning types was not statistically different between tonic and phasic neurons ($X_{5}^{2}$ = 7.97, *p* > 0.1).

**FIGURE 3:**
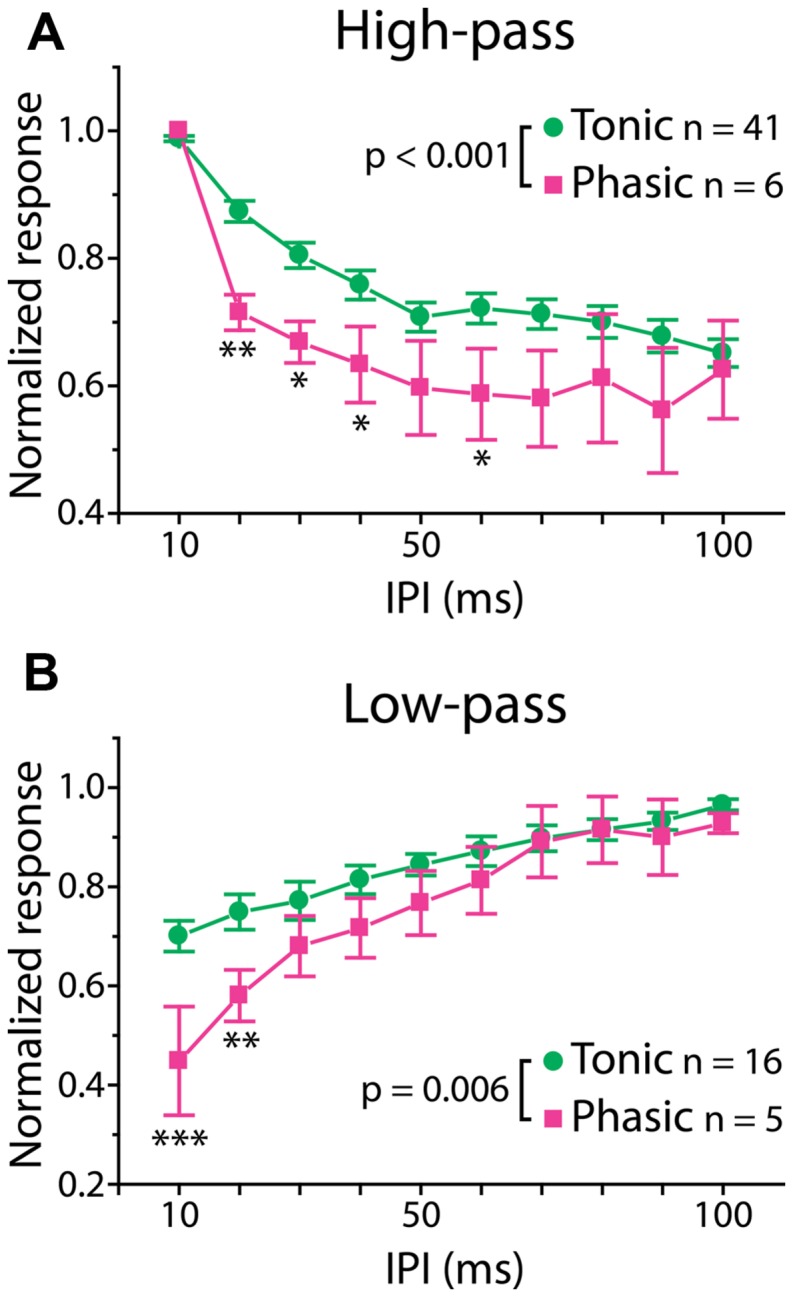
**Phasic neurons exhibit sharper synaptic IPI tuning than tonic neurons.** Averaged IPI tuning curves for synaptic potentials obtained from tonic (green) and phasic (magenta) neurons. Two-way repeated measures ANOVA revealed a significant interaction effect between firing type and IPIs for both high-pass (**A**; *p* < 0.001) and low-pass (**B**; *p* = 0.006) responses. Compared to tonic neurons, phasic neurons exhibited high-pass tuning curves that tended to drop sharply at 20 ms and remain low toward longer IPIs, and low-pass tuning curves with a larger drop at short IPIs. Single, double and triple asterisks represent statistical significance of *p* < 0.05, *p* < 0.01, and *p* < 0.001, respectively (tested by the *post hoc* Holm–Sidak method at each IPI).

Interestingly, however, there was a significant correlation between firing pattern and the shape of PSP tuning curves, which represents temporal filtering of subthreshold synaptic inputs. Here, we focused on two major tuning types, high-pass and low-pass tuning, which comprised 58% of our tuning curves. As illustrated by averaged PSP tuning curves in **Figure 3** (see also **Figure 1**, right panels, for individual tuning curves), phasic neurons exhibited a sharper decrease in synaptic response compared to tonic neurons. A two-way repeated-measures ANOVA revealed a highly significant interaction effect between firing pattern and IPI in both tuning types [high-pass: *F*_(9,405)_ = 3.44, *p* < 0.001, low-pass: *F*_(9,171)_ = 2.67, *p* = 0.006]. A *post hoc* multiple comparison analysis (Holm–Sidak method) revealed that the normalized responses of phasic neurons are significantly reduced at 20–40 and 60 ms IPIs (*p* < 0.05) for high-pass tuning and at 10 and 20 ms IPIs for low-pass tuning (*p* < 0.01). These results suggest that, in ELp neurons, intrinsic membrane properties that determine spike frequency adaptation do not only influence the PSP-to-spike conversion in response to suprathreshold input, but also affect subthreshold responses to synaptic input.

### TONIC NEURONS HAVE SHARPER ACTION POTENTIALS THAN PHASIC NEURONS

**FIGURE 4:**
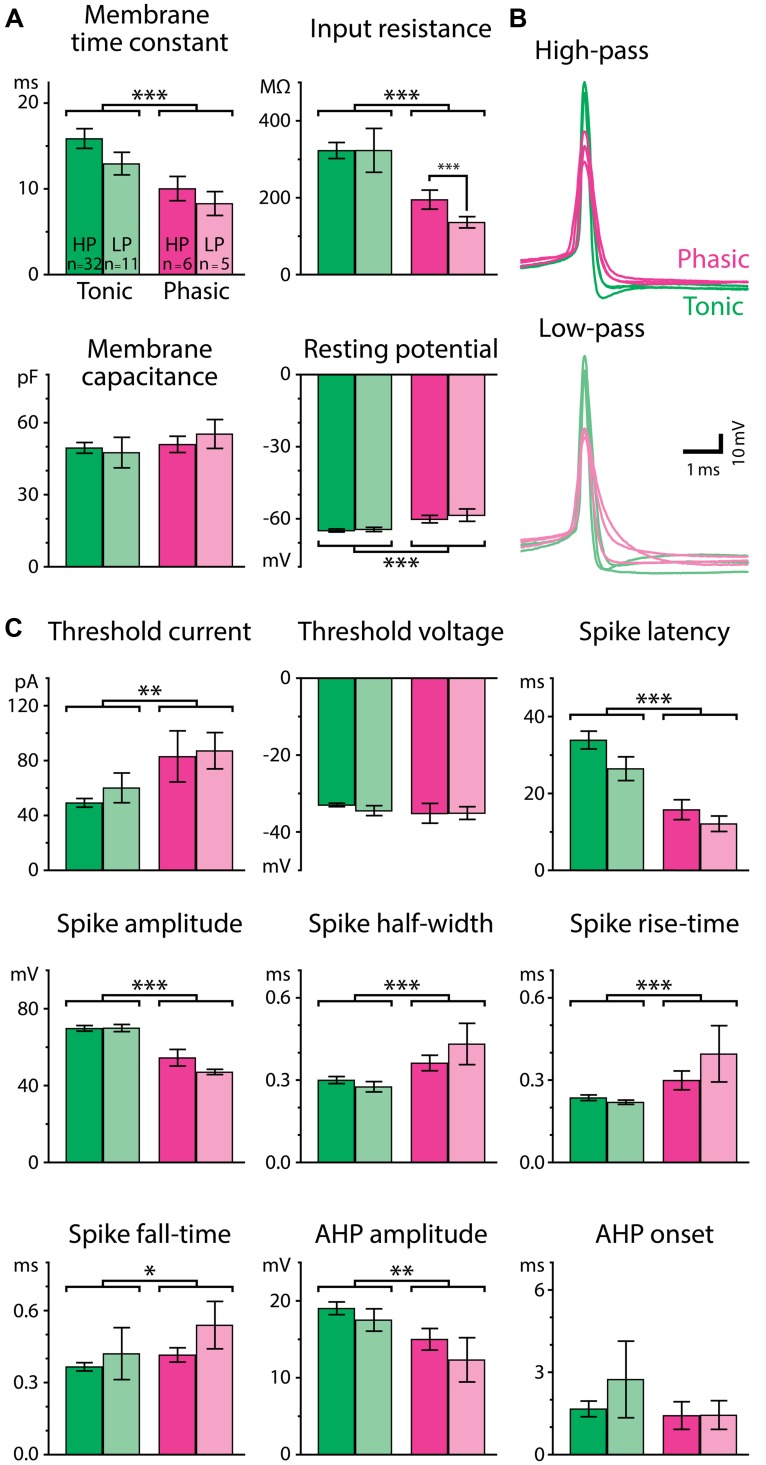
**Passive and active membrane properties differ for tonic and phasic neurons. (A)** Resting membrane properties of tonic (green bars) and phasic (magenta bars) neurons obtained from voltage responses to small hyperpolarizing current steps (–3 to –20 pA). Neurons were further categorized by the type of synaptic IPI tuning exhibited by the cell (High-pass; HP; vivid-color bars, Low-pass; LP; pale-color bars). Tonic neurons had a longer membrane time constant, larger input resistance, and more hyperpolarized resting potential than phasic neurons (two-way ANOVA followed by the Holm–Sidak multiple comparison analysis, significant differences between groups are indicated by parenthesis and asterisks). Single, double, and triple asterisks represent *p* < 0.05, *p* < 0.01, and *p* < 0.001, respectively. **(B)** Superimposed traces of action potentials induced by threshold current injection from six phasic and six tonic neurons. Traces are aligned in time at the action potential peak and in voltage at the action potential threshold. Among neurons having either high-pass (top; vivid-color) or low-pass responses (bottom; pale-color), action potentials of tonic neurons (green) had larger amplitudes and shorter durations compared to phasic neurons (magenta). **(C)** Parameters of action potentials. See Materials and Methods for details of the measurements. Color scheme of the bars and the number of neurons within each category are the same as in **(A)**.

To understand how the excitability of postsynaptic neurons shapes the temporal filtering of IPIs in ELp, we characterized intrinsic electrophysiological properties of tonic and phasic neurons. Forty-three tonic and eleven phasic neurons that exhibited either high-pass or low-pass tuning were used to investigate membrane properties and spike parameters. We first focused on the difference in resting membrane properties between tonic and phasic neurons (**Figure 4A**). Tonic neurons exhibited more hyperpolarized resting membrane potential [*F*_(1,50)_ = 17.05, *p* < 0.001, two-way ANOVA, the same test applies below], larger membrane time constant [*F*_(1,50)_ = 12.49, *p* < 0.001], and larger input resistance [*F*_(1,50)_ = 12.54, *p* < 0.001] than phasic neurons. However, membrane capacitance was similar between tonic and phasic neurons [*F*_(1,50)_ = 0.83, *p* > 0.3], suggesting that tonic and phasic neurons are similar in size but express different sets of ionic conductances at rest.

Tonic and phasic neurons were also distinguishable by the waveform of individual action potentials (**Figures 4B,C**). Tonic neurons generated larger [spike amplitude; *F*_(1,50)_ = 48.36, *p* < 0.001] and faster action potentials as seen in shorter half-width [*F*_(1,50)_ = 14.58, *p* < 0.001], rise-time [*F*_(1,50)_ = 14.09, *p* < 0.001], and fall-time [*F*_(1,50)_ = 4.64, *p* = 0.036], compared to phasic neurons. The AHP was also larger in tonic neurons [AHP amplitude; *F*_(1,50)_ = 7.66, *p* = 0.008], although the time to the trough of the AHP (AHP onset) was not different between tonic and phasic neurons [*F*_(1,50)_ = 0.00, *p* > 0.9]. In addition, tonic neurons required less current to fire an action potential [threshold current; *F*_(1,50)_ = 10.36, *p* = 0.002], whereas their threshold membrane potential was not different from that of phasic neurons [threshold voltage; *F*_(1,50)_ = 1.25, *p* > 0.2]. Spike latency at threshold current was longer in tonic neurons than in phasic neurons [*F*_(1,50)_ = 29.09, *p* < 0.001]. The difference in threshold current and spike latency was consistent with the high input resistance and long membrane time constant of tonic neurons.

Since resting properties and spike shape have been reported to correlate with the tuning of synaptic responses in other systems (reviewed in O’Donnell and Nolan, 2011), we further considered IPI tuning categories (high-pass or low-pass) in our analysis of resting membrane properties and spike waveform. Among thirteen parameters we measured, input resistance was the only variable characterized by a significant interaction effect between firing pattern and tuning type [*F*_(1,50)_ = 9.44; *p* = 0.003, other parameters: *p* > 0.1]. In particular, high-pass phasic neurons had significantly higher input resistance than low-pass phasic neurons (Holm–Sidak *post hoc* analysis; *t* = 3.54, *p* < 0.001), suggesting that resting ionic conductance contributes to IPI tuning type, at least among phasic neurons. Overall, however, the general lack of a correlation between intrinsic properties and tuning type implies that synaptic mechanisms (George et al., 2011; Baker et al., 2013; Ma et al., 2013) contribute mainly to determining gross tuning type, e.g., high-pass vs. low-pass, and that intrinsic properties rather contribute to fine adjustments of IPI tuning.

### Ni^2+^-SENSITIVE CURRENTS ARE TIGHTLY RELATED TO PHYSIOLOGICAL DIFFERENCES BETWEEN TONIC AND PHASIC NEURONS

**FIGURE 5:**
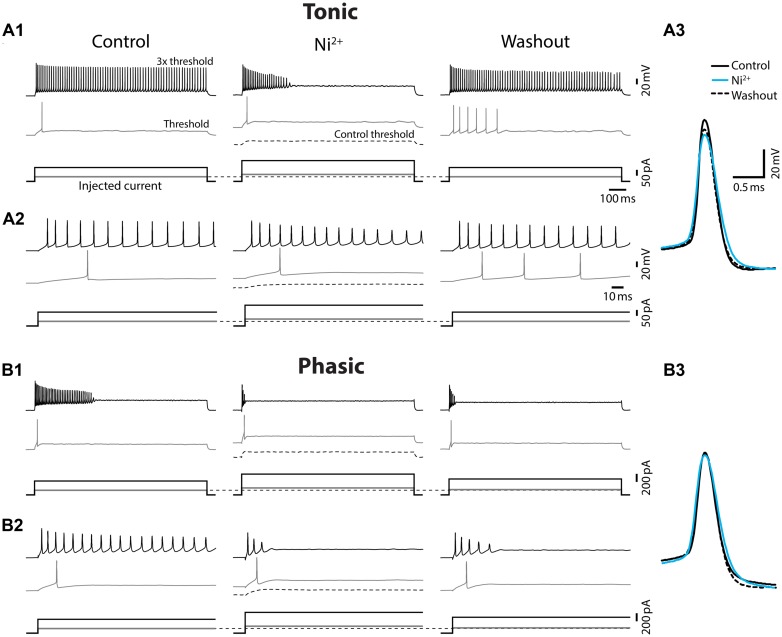
**Ni^2+^ application changed tonic firing mode into phasic.** Spiking responses of a tonic **(A)** and a phasic **(B)** neuron to depolarizing current injection before (control), during (Ni^2+^), and after (washout) bath application of the Ca^2+^ channel blocker, Ni^2+^. All scale bars and legends, except for the vertical bar in the current waveforms, are shared between **(A)** and **(B)**. **(A1)** Upper panel, Spiking responses obtained with threshold (gray trace) and three-time threshold (black trace) current injection. Corresponding current waveforms are shown in lower panel. Ni^2+^ application increased threshold current (see no spiking at control threshold current, black dotted lines) and changed the firing pattern of this tonic neuron into phasic. Threshold current and tonic firing behavior were recovered by washout. Initial response traces for 150 ms after current onset are shown in **(A2)**. **(A3)** Superimposed traces of action potentials induced by threshold current injection. Traces are aligned at peak time and threshold voltage. Ni^2+^ application (cyan) reduced the amplitude and increased the width of action potentials from control (black). The effects recovered, at least partially, after washout (black dotted line). **(B)** Ni^2+^ application further facilitated spike frequency adaption and increased the threshold current intensity of the phasic neuron (**B1** and initial 150 ms response** B2**), but effects on spike waveform were much smaller compared to tonic neurons **(B3)**.

**FIGURE 6:**
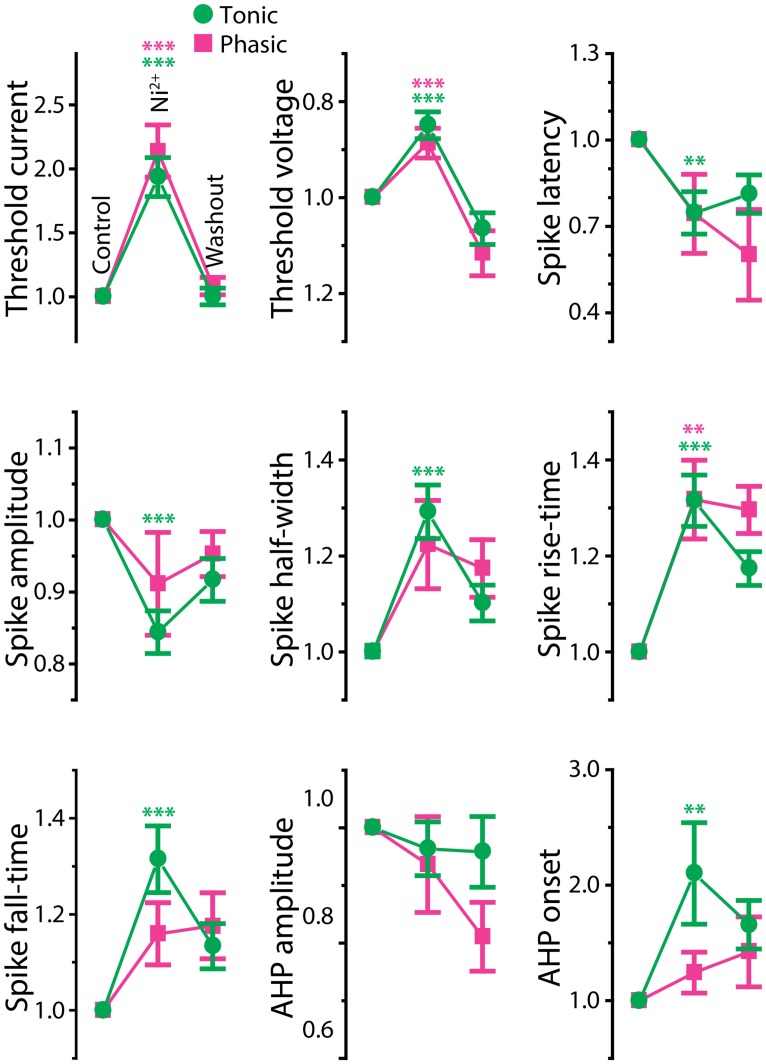
**Effects of Ni^2+^ on spike shape of tonic and phasic neurons.** Spike parameters of tonic (green circle) and phasic (magenta square) neurons before (control; left), during (Ni^2+^; middle) and after (washout; right) Ni^2+^ application. Although measurements are reported as relative value to control, statistical significance was evaluated using raw values. Asterisks indicate significant difference between control and Ni^2+^ (green: tonic neurons, magenta: phasic neurons; double and triple asterisks represent *p* < 0.01 and *p* < 0.001, respectively, Holm–Sidak multiple comparison test following one-way repeated measures ANOVA). Ni^2+^ application significantly reduced spike amplitude and increased spike duration (half-width, rise-time, fall-time, and AHP onset) in tonic neurons. In phasic neurons, Ni^2+^ also increased spike rise-time; however, it did not significantly affect spike amplitude, fall-time, half-width, and AHP onset. Note that the treatment increased threshold current and depolarized threshold voltage similarly in tonic and phasic neurons.

Ca^2+^-activated potassium (K_Ca_) currents can play critical roles in regulating spike frequency adaptation, sharpening of action potentials by facilitating repolarization, and mediating AHPs (reviewed in Sah, 1996; Sah and Faber, 2002). These effects are similar to the observed differences between tonic and phasic ELp neurons. To test the role of K_Ca_ currents in shaping divergent electrophysiological properties of ELp neurons, we bath applied the Ca^2+^ channel blocker Ni^2+^ at a high concentration that effectively blocks all Ca^2+^ channels regardless of channel subtypes (1–3 mM; “non-specific concentration:” Magee and Carruth, 1999; **Figure 5** and summarized **Figure 6**). Twenty tonic neurons were subjected to Ni^2+^ application (Ni^2+^) and 15 of these were further tested for washout to verify specific effects of Ni^2+^ (washout). In most cases, Ni^2+^ application changed the tonic firing mode into phasic (14 out of 20), and the firing pattern returned to tonic after washout (8 out of 10 neurons which were tested for washout after a changing firing pattern in response to Ni^2+^ treatment; **Figures 5A1,A2**), indicating that spike frequency adaptation is suppressed in tonic neurons by Ni^2+^-sensitive currents. In addition to the firing pattern, many physiological properties of tonic neurons that differ from phasic neurons were sensitive to Ni^2+^ and the effects were reversible by washout, suggesting that Ni^2+^-sensitive currents play critical roles in establishing the intrinsic excitability characteristic of tonic neurons: Ni^2+^ application increased the spike threshold current (**Figures 5A1,A2**, bottom, see also threshold current in **Figure 6**, *t* = 7.04, *p* < 0.001, Holm–Sidak multiple comparison analysis between Control and Ni^2+^ following one-way repeated measures ANOVA, measured after 2 min of Ni^2+^ wash-in or washout, the same test applies below) and depolarized the threshold voltage (**Figure 6**, *t* = 6.62, *p* < 0.001), suggesting blockade of voltage-gated Ca^2+^ (Ca_v_) channels that supply inward current during subthreshold depolarization. The decrease in spike latency (**Figure 5A2**, gray trace; **Figure 6**, *t* = 3.72, *p* = 0.002) may result from the lack of a voltage-dependent increase in membrane time constant that is associated with open Ca_v_ channels. As shown in **Figure 5A3**, the evoked action potential after Ni^2+^ wash-in showed decreased spike amplitude (**Figure 6**, *t* = 5.56, *p* < 0.001) and increased spike width (**Figure 6**, half-width: *t* = 6.34, *p* < 0.001, rise-time: *t* = 7.25, *p* < 0.001, and fall-time: *t* = 6.42, *p* < 0.001). Ni^2+^ wash-in also significantly increased AHP onset (**Figure 6**, *t* = 3.56, *p* = 0.004) but did not affect AHP amplitude (**Figure 6**, *t* = 1.00, *p* > 0.5). Despite the drastic effects of Ni^2+^ application on active membrane properties, the treatment did not affect resting membrane potential (*t* = 1.63, *p* > 0.3), nor passive membrane properties (membrane time constant, membrane capacitance, and input resistance; *t* = 0.45, 2.28, and 1.19, respectively, *p* > 0.1, eight cells).

Ni^2+^ application to phasic neurons further promoted the phasic firing pattern, but had less impact on spike shape compared to tonic neurons (Ni^2+^; seven cells, washout; five cells; **Figure 5B**): among nine parameters compared in **Figure 6**, only threshold current, threshold voltage and spike rise-time exhibited a significant change during Ni^2+^ wash-in in the same direction as tonic neurons (*t* = 5.24, *p* < 0.001, *t* = 6.13, *p* < 0.001, and *t* = 4.01, *p* = 0.007, respectively). None of the other spike parameters (spike latency, spike amplitude, half-width, and fall-time; *t* = 1.84, 1.77, 2.66, and 2.42, respectively, *p* > 0.05) including AHP (amplitude and onset; *t* = 1.69 and 0.73, respectively, *p* > 0.3), resting potential (*t* = 0.67, *p* > 0.5), and passive membrane properties (membrane time constant, capacitance, and input resistance; *t* = 0.57, 0.64 and 1.32, respectively, *p* > 0.5, four cells) were significantly affected.

The effects of Ni^2+^ application on tonic neurons, particularly in spike fall-time and AHP onset, suggests that repolarization of action potentials is facilitated by an outward current that is sensitive to Ni^2+^, indicative of a K_Ca_ current. Thus, we hypothesized that a K_Ca_ current in tonic neurons rapidly repolarizes the membrane potential during spikes, quickly releases the inactivation of sodium channels and thus suppresses spike frequency adaptation. In phasic neurons, by contrast, no effect of Ni^2+^ on spike repolarization suggests that the K_Ca_ current is not as large as in tonic neurons. However, Ni^2+^ did affect spike threshold similarly in both tonic and phasic neurons, suggesting that Ca_v_ channels are activated by subthreshold depolarization to regulate spike threshold in both types of neurons.

### TONIC NEURONS EXPRESS LARGER FAST Ni^2+^-SENSITIVE OUTWARD CURRENTS

**FIGURE 7:**
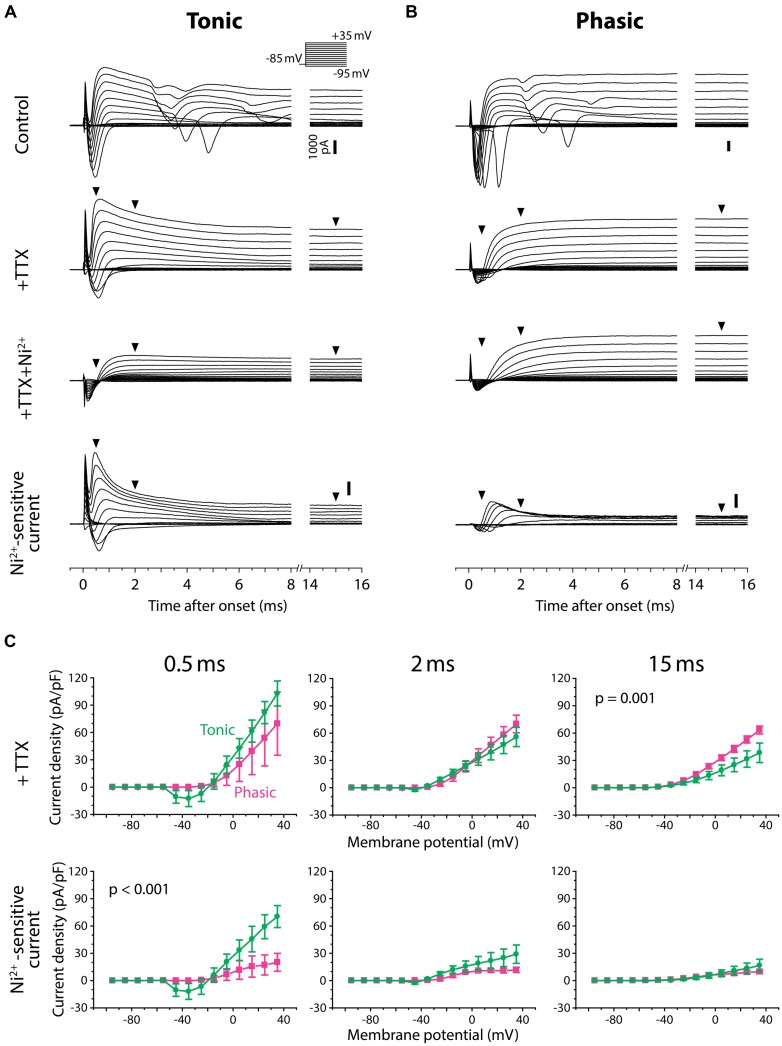
**Tonic neurons exhibit a larger Ni^2+^-sensitive outward current than phasic neurons. (A,B)** Voltage-clamp recordings from a tonic neuron **(A)** and a phasic neuron **(B)** during voltage steps from a holding potential of –85 mV to test potentials between –95 and +35 mV in 10 mV increments [inset in **(A)**]. Neurons were tested before (control), after application of tetrodotoxin (+TTX), and after combined application of TTX and Ni^2+^ (+TTX+Ni^2+^). +TTX+Ni^2+^ currents were subtracted from +TTX currents to obtain the Ni^2+^-sensitive currents (bottom). Both tonic and phasic neurons in the control condition showed multiple spikes of inward current in response to test potentials depolarized above –25 mV. The spikes were abolished by TTX, suggesting sodium spikes resulting from a poor space clamp. Vertical scale bars (1000 pA) shown in control are also applied to +TTX and +TTX+Ni^2+^ currents from the same cell. Bottom, Ni^2+^-sensitive currents consisted of a fast inward current and an outward current, suggesting that the former was a voltage-gated Ca^2+^ (Ca_v_) current and the latter was a Ca^2+^-activated K^+^ (K_Ca_) current elicited by the Ca_v_ current. Note, in the tonic neuron, that the fast transient outward component observed during +TTX application was largely blocked by Ni^2+^. Arrowheads indicate where the current amplitude was measured in **(C)** (0.5, 2, and 15 ms after the onset of voltage steps). **(C)** Current–voltage relationship of +TTX current (top) and Ni^2+^-sensitive current (bottom) at the times indicated. Current density (current amplitude divided by resting membrane capacitance) obtained from three tonic (green circle) and three phasic (magenta square) neurons was plotted against test potential. To remove capacitive currents due to incomplete capacitance and series resistance compensation, we considered current responses between –95 and –75 mV as passive, and used these to generate a linear estimate of the passive current response at each test potential, which we subtracted from the raw data before averaging (see Materials and Methods for details). *p* value indicates a significant interaction effect (*p* < 0.05) between voltage and firing pattern (evaluated by two-way repeated measures ANOVA). A fast-decaying outward Ni^2+^-sensitive current (at 0.5 ms) was larger in tonic neurons than phasic neurons particularly at test potentials depolarized to –5 mV. Note that the putative transient Ca_v_ current was observed at potentials as low as –45 mV (at 0.5 ms), which is below the spike threshold voltage measured in current-clamp **(Figure 4)**. Also note that amplitude of the Ni^2+^-sensitive steady-state outward current (at 15 ms) was not significantly different between tonic and phasic neurons, but this late component in the +TTX current was larger in phasic neurons than tonic neurons.

To test for Ni^2+^-sensitive currents in tonic and phasic neurons, we performed voltage-clamp recordings from three tonic neurons and three phasic neurons and examined the voltage-dependence as well as kinetics of Ni^2+^-sensitive currents (**Figure 7**). The membrane potential of recorded neurons was stepped from a holding potential of –85 mV to test potentials of –95 mV to + 35 mV in 10 mV increments. As they were depolarized, all tonic and phasic neurons in normal ACSF showed a spike of inward current at voltages ranging from –55 to –35 mV, and multiple spikes at more depolarized potentials (**Figures 7A,B**; control). The spikes were eliminated by adding the Na^+^ channel blocker TTX (1 μM) to ACSF (+TTX), demonstrating that the multiple spikes were sodium action potentials resulting from a poor space clamp likely due to the multipolar morphology of ELp neurons (Xu-Friedman and Hopkins, 1999; George et al., 2011; Ma et al., 2013). We determined the sensitivity of the remaining inward and outward currents to Ni^2+^ by further applying Ni^2+^ in the presence of TTX (+TTX+Ni^2+^), and then isolated Ni^2+^-sensitive currents by subtracting +TTX+Ni^2+^ currents from +TTX currents. Finally, we summarized the current–voltage relationship across neurons (**Figure 7C**). To account for leak conductance and capacitive current resulting from poor series resistance and capacitance compensation, conductances measured between –95 and –75 mV were subtracted from the traces before analysis (see Materials and Methods for details). The compensated currents were further divided by membrane capacitance (current density) for comparison. This method could not fully account for poor space clamp, incomplete series resistance compensation and leak conductances, which could lead to an underestimation of whole-cell currents. Nevertheless, this method did allow us to qualitatively assess the presence of inward and outward currents, as well as detect differences between tonic and phasic neurons.

As we depolarized the test potential, every tonic and phasic neuron exhibited a voltage-dependent outward current that was sensitive to Ni^2+^, indicating a K_Ca_ current activated by Ca^2+^ influx (**Figures 7A,B**, Ni^2+^-sensitive current). The current consisted of a fast-activating transient component with a peak occurring at 0.5–1.0 ms after the step onset and a sustained component. As summarized in **Figure 7C** (bottom panels), the fast transient Ni^2+^-sensitive outward current was larger in tonic neurons than in phasic neurons, particularly at around the peak and above –5 mV [interaction between firing type and membrane potential: at 0.5 ms after step onset; *F*_(13,52)_ = 3.56, *p* < 0.001, at 2 ms; *F*_(13,52)_ = 1.78, *p* > 0.05, two-way repeated measures ANOVA]. Amplitude of the sustained component was, however, not different between tonic and phasic neurons [at 15 ms; *F*_(13,52)_ = 0.41, *p* > 0.9]. These results suggest that high expression of the fast transient K_Ca_ current contributes to the sharp spikes of tonic neurons via its rapid activation during action potentials. The results also imply that the transient K_Ca_ current in phasic neurons is not large enough to suppress accumulation of sodium channel inactivation, leading to strong spike frequency adaptation.

After TTX application, all tonic and phasic neurons also exhibited a voltage-dependent inward current at test potentials more positive than –55 to –35 mV (**Figures 7A,B**, +TTX), which was below the spike threshold voltage determined in current-clamp (see **Figure 4C**, threshold voltage). This inward current was completely blocked by adding Ni^2+^ (**Figures 7A,B**, +TTX+Ni^2+^ and Ni^2+^-sensitive current), indicating a Ca_v_ current. Within the Ni^2+^-sensitive current, the inward component was always followed by an outward component, suggesting activation of K_Ca_ current by the Ca^2+^ current. These observations further suggest a role of K_Ca_ channels in shaping PSPs by adding membrane conductances in a depolarization- and Ca^2+^-dependent manner.

In the TTX-only perfusate (before adding Ni^2+^), the amplitude of the early outward component was similar across test potentials between tonic and phasic neurons [**Figure 7C**, +TTX: at 0.5 ms; *F*_(13,52)_ = 1.81, *p* > 0.05, at 2 ms; *F*_(13,52)_ = 1.63, *p* > 0.1]. However, the stable outward current in this condition was significantly larger in phasic neurons than in tonic neurons [**Figure 7C**, +TTX: at 15 ms, *F*_(13,52)_ = 3.26, *p* = 0.001]. This difference in the stable component reflects the difference in +TTX+Ni^2+^ outward current that was observed at test potentials more positive to –45 mV (**Figures 7A,B**): the amplitude of +TTX+Ni^2+^ current was larger in phasic neurons than in tonic neurons at 2 ms [*F*_(13,52)_ = 3.06, *p* = 0.002] and 15 ms [*F*_(13,52)_ = 28.25, *p* < 0.001]. Compared to the putative K_Ca_ current, activation of +TTX+Ni^2+^ current was slower; the peak occurred >0.9 ms after onset and the amplitude was not different between tonic and phasic neurons at 0.5 ms [*F*_(13,52)_ = 0.09, *p* > 0.9]. Outward +TTX+Ni^2+^ current was TTX-insensitive and Ni^2+^-insensitive, suggesting a Ca^2+^-independent and depolarization-activated K^+^ (K_v_) current. Indeed, the general K_v_ blocker 4-AP (1 mM, added to +TTX condition, data not shown) blocked the slow outward current of both tonic and phasic neurons [4-AP-senstive current evoked by a test pulse of +35 mV, measured at 15 ms; three tonic and one phasic neurons, 27.6 ± 1.3 pA/pF and 44.9 pA/pF, respectively]. These results suggest that phasic neurons express larger low-voltage-gated and sustained K_v_ current. The slow-inactivation of this K_v_ current possibly provides a long-lasting outward current during synaptic depolarization and suppresses repetitive firing. Thus, in addition to fast spike frequency adaptation resulting from small K_Ca_ currents, K_v_ currents may suppress tonic firing in phasic neurons.

### LARGE-CONDUCTANCE K_Ca_ CURRENTS MEDIATE TONIC FIRING

**FIGURE 8:**
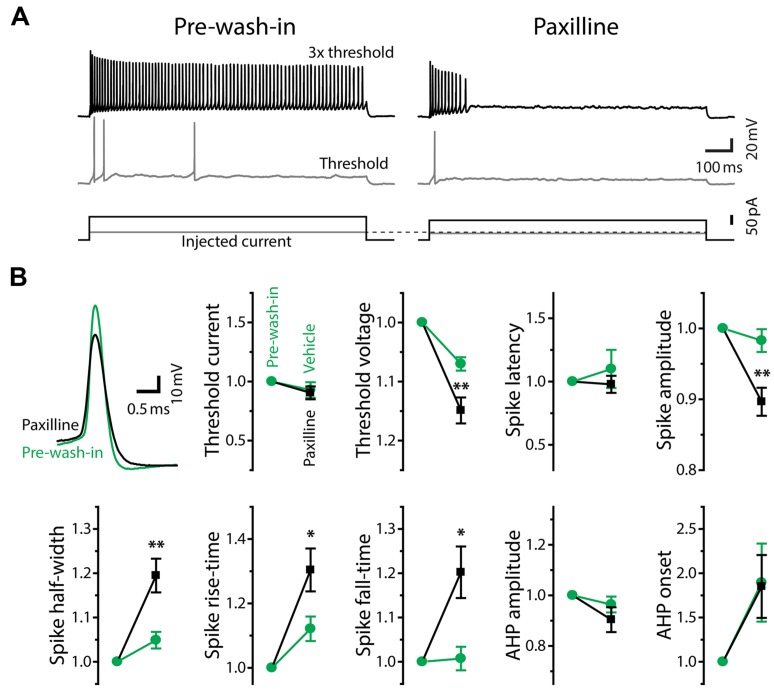
**Large-conductance Ca^2+^-activated potassium currents mediate tonic firing. (A)** Firing mode of a tonic neuron changed from tonic (left, pre-wash-in) to phasic (right) during bath application of the large-conductance K_Ca_ (BK) channel blocker, paxilline (after 10 min of wash-in). Traces are labeled as in **Figure 5A**. Note that, unlike Ni^2+^ application **(**Figure 5**)**, paxilline did not increase threshold current intensity. **(B)** Upper left, superimposed traces of single action potentials induced by threshold current injection, aligned at peak time and threshold voltage. Paxilline application (black) reduced the amplitude and increased the width of action potentials (pre-wash-in, green). Traces are obtained from the same cell in **(A)**. Graphs, spike parameters after 8–12 min wash-in of paxilline (black) or DMSO (vehicle, green). Measurements are reported as relative to those before wash-in (pre-wash-in, green). Single and double asterisks represent significant difference (*p* < 0.05, and *p* < 0.01, respectively, evaluated by Student’s *t*-test) between relative changes during paxilline and vehicle application. Although there were slight changes during vehicle wash-in, suggesting a non-specific effect of long-term recording, paxilline further lowered spike amplitude, increased spike rise-time, fall-time, and half-width, similar to Ni^2+^ application. However, unlike Ni^2+^, paxilline did not significantly affect threshold current and AHP, and it significantly hyperpolarized threshold voltage.

**FIGURE 9:**
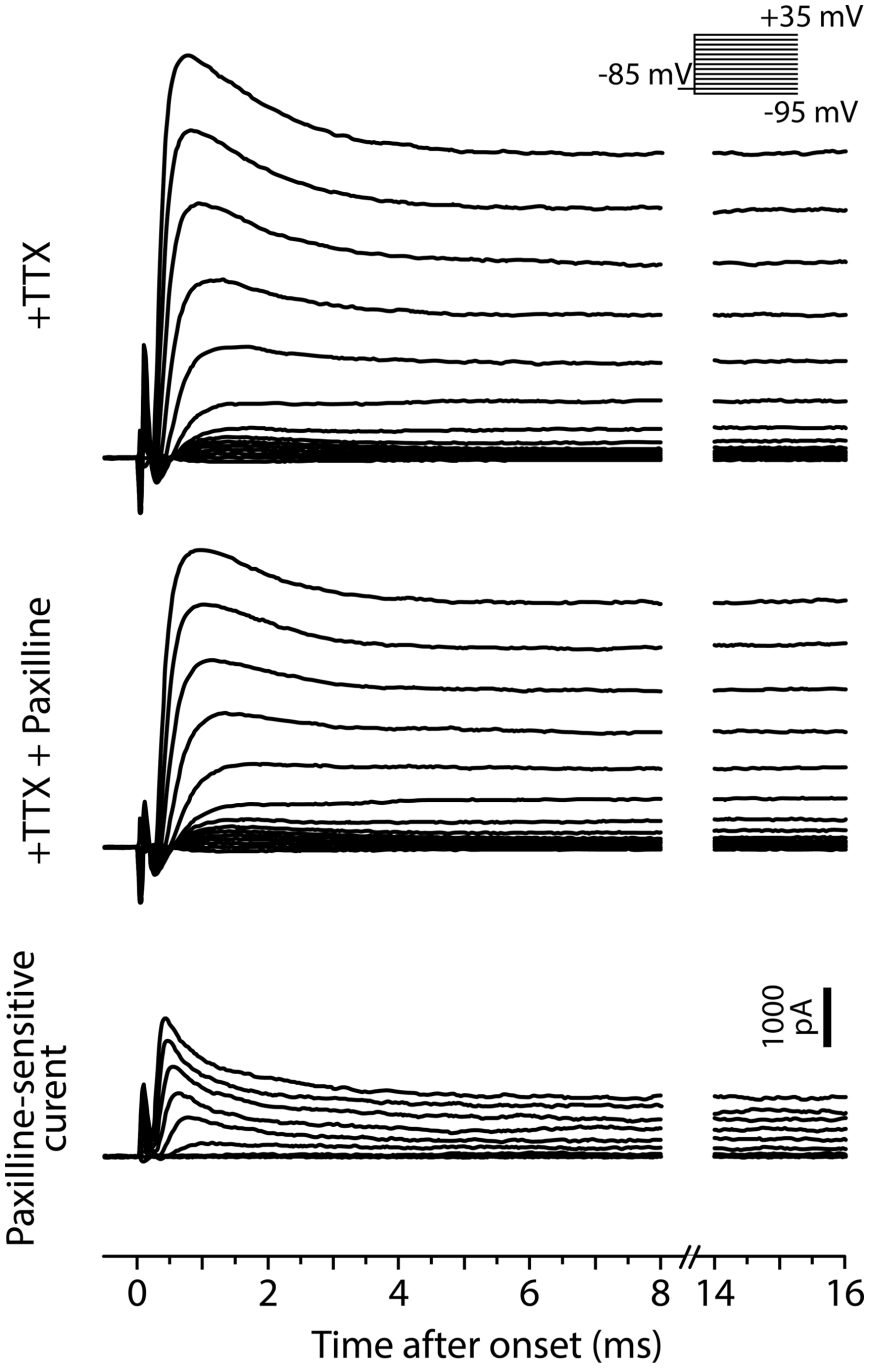
**Fast BK current in tonic neurons.** Voltage-clamp recordings from a tonic neuron during TTX application (+TTX) and following application of paxilline combined with TTX (+TTX+Paxilline, after 5 min of wash-in). Inset, the neuron was tested with the same voltage steps as in **Figure 7**. Paxilline-sensitive currents (bottom), suggesting BK currents, were obtained by subtracting +TTX+Paxilline currents from +TTX currents. The BK currents exhibited a fast and partially inactivating component.

Finally, we pharmacologically identified the subtype of K_Ca_ channels underlying tonic firing. Increased spike fall-time during Ni^2+^ application and kinetics of the fast Ni^2+^-sensitive outward current, particularly in tonic neurons, are consistent with a contribution from large-conductance K_Ca_ channels, or BK channels (effects on spike waveform: Sah and Faber, 2002, current kinetics: e.g., Behrens et al., 2000). Furthermore, recent studies have shown that BK currents oppose spike frequency adaptation (Gu et al., 2007; Perez et al., 2013). To examine whether BK currents are indeed responsible for sharp spike shape, tonic firing pattern and fast Ni^2+^-sensitive currents in tonic neurons, we bath-applied the BK-specific channel blocker, paxilline (10 μM in 0.04% DMSO in ACSF), to tonic neurons under current- and voltage-clamp (**Figures 8** and **9**).

In response to depolarizing current injections, paxilline application facilitated spike frequency adaptation in all of sixteen tonic neurons, and twelve of them eventually reached a phasic firing pattern after 4–29 min of wash-in (average: 14.1 ± 2.6 min; **Figure 8A**). Because application of vehicle alone (0.04% DMSO in ACSF) never changed the firing pattern (11 tonic neurons, wash-in duration: 9.5–45.5 min; average: 24.1 ± 2.9 min), we concluded that the change in firing pattern was due to a specific blockade of BK channels. Paxilline application also changed the waveform of individual action potentials from a tonic-typical shape to a phasic-typical shape: the drug decreased the amplitude and increased the duration (**Figure 8B**, upper left). In **Figure 8B**, we show action potential parameters obtained after 8–12 min of wash-in. We evaluated specific effects of paxilline by comparing relative changes during paxilline application (16 cells) to non-specific changes during vehicle application (11 cells). Similar to the effect of Ni^2+^ application (**Figure 6**), paxilline significantly reduced spike amplitude (*t* = 3.16, *p* = 0.004) and increased spike half-width (*t* = 3.0, *p* = 0.006), rise-time (*t* = 2.11, *p* = 0.045), and fall-time (*t* = 2.64, *p* = 0.014). These changes parallel the observed intrinsic differences between tonic and phasic neurons (**Figure 4**), although paxilline did not significantly affect the AHP (onset and amplitude: *t* = 0.30 and 1.46, respectively, *p* > 0.1), resting membrane properties (resting potential, membrane time constant, membrane capacitance and input resistance: *t* = 1.21, 0.83, 0.85, and 0.46, respectively, *p* > 0.2) nor spike latency (*t* = 0.82, *p* > 0.4). Notably, unlike Ni^2+^ application which increased spike threshold current and depolarized threshold voltage, paxilline had no significant effect on threshold current (*t* = 0.28, *p* > 0.7) but significantly hyperpolarized threshold voltage (*t* = 2.79, *p* = 0.01), suggesting that BK currents are activated during subthreshold depolarization and counteract subthreshold Ca_v_ currents to determine threshold voltage. Activation of BK conductance prior to action potential initiation is also consistent with the observed elongation of spike rise-time, not only fall-time, by paxilline. Reduced spike amplitude during paxilline application may also be explained, at least partially, by this early BK activation: the slow rising phase of spikes may allow slower K_v_ currents to become activated closer to the spike peak, thereby truncating the action potential. Indeed, fast BK currents indirectly limit participation of K_v_ channels during action potentials by modifying spike shape in anterior pituitary cells (Van Goor et al., 2001).

We also recorded paxilline-sensitive currents from two tonic neurons. **Figure 9** exemplifies voltage-dependent current responses obtained from one of the neurons in response to the same voltage-step protocol used in **Figure 7**. We first bath-applied TTX (+TTX) and then switched to perfusate containing TTX and paxilline (+TTX+Paxilline, wash-in duration: 5 min) to obtain paxilline-sensitive current by subtracting +TTX+Paxilline currents from +TTX currents. Both of the two neurons exhibited fast-activating and partially inactivating outward currents, similar to the Ni^2+^-sensitive outward currents of tonic neurons. Overall, these results suggest that fast BK current is essential to establishing the membrane excitability that typifies tonic neurons in response to both sub- and suprathreshold depolarizations.

## DISCUSSION

**FIGURE 10:**
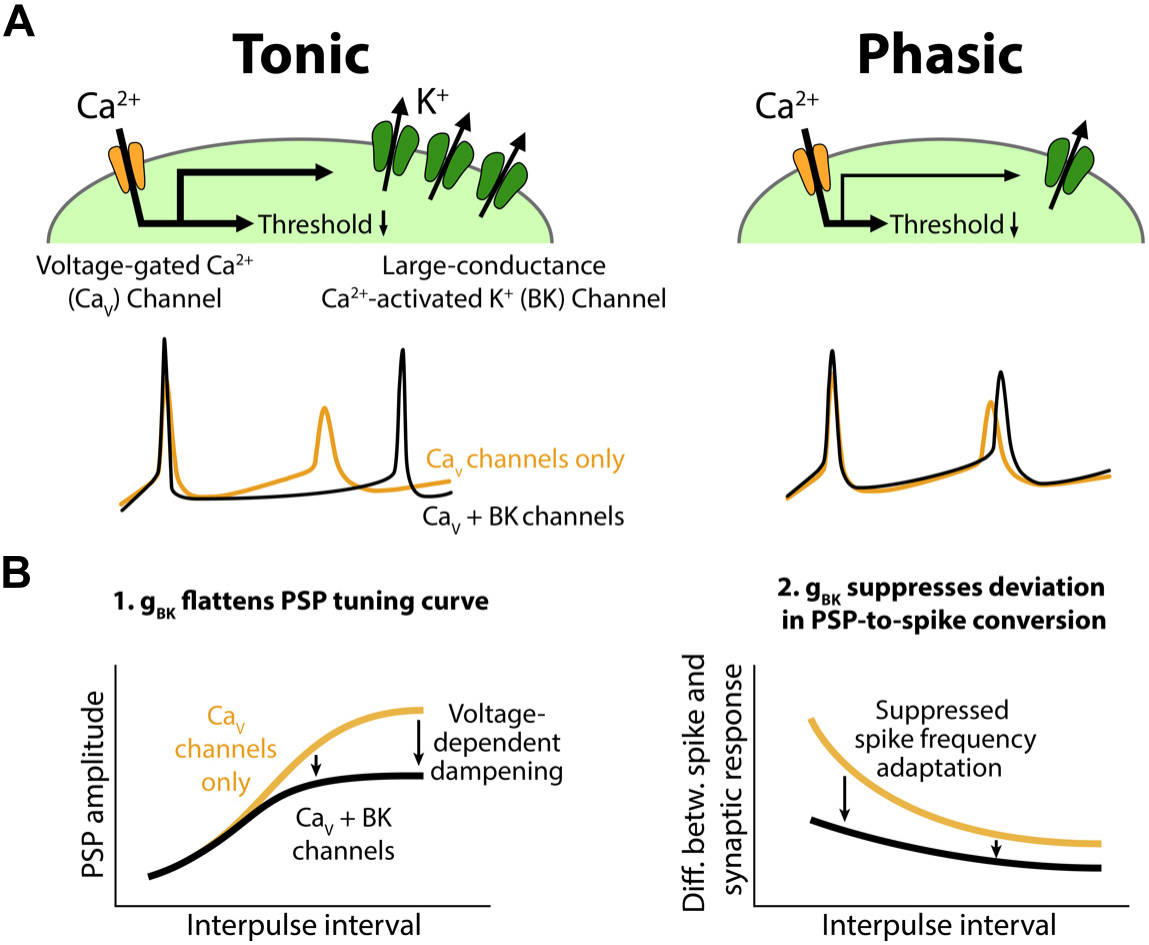
**Conceptual model for the role of BK channels in shaping interval tuning. (A)** Top, Both tonic (left) and phasic (right) ELp neurons express Ca_v_ channels and BK channels. BK current density is larger in tonic neurons. Synaptic depolarization activates the Ca_v_ channels below spike threshold, resulting in a reduction in the synaptic current required to induce action potentials. Bottom, ELp neurons show strong spike frequency adaptation without BK currents (yellow; Ca_v_ channels only). Fast BK current activated by the Ca^2+^ influx during action potentials accelerates the membrane repolarization and limits sodium channel inactivation to promote repetitive spiking (black; Ca_v_ + BK channels), particularly in tonic neurons (left). Due to the small BK current, phasic neurons still show significant spike frequency adaptation (right). **(B)** Models explaining how BK conductance (g_BK_) regulates IPI tuning curves for PSP amplitude (left) and spike number (right). Left, Voltage-dependent Ca^2+^ influx during synaptic depolarization activates subthreshold g_BK_ in a depolarization-dependent manner. Thus, the larger the synaptic excitation is, the more the resulting PSP is attenuated by the g_BK_ (arrows) so that the overall shape of the tuning curve is flattened. Right, BK current allows neurons to follow stimulus trains with spikes by suppressing spike frequency adaptation. Thus, BK current suppresses the difference between IPI tuning curves that occurs when synaptic input is converted into spike output. This effect is larger at short IPIs compared to long IPIs (arrows) because synaptic responses summate at short IPIs, resulting in large and prolonged excitatory PSPs.

We found variation in spike frequency adaptation among ELp neurons that correlated with IPI tuning to afferent input. Strongly adapting (phasic) neurons exhibited sharper IPI tuning than weakly adapting (tonic) neurons. Tonic neurons also converted synaptic responses into spike output more faithfully than phasic neurons. Pharmacological and voltage-clamp evidence suggests that tonic neurons express a fast, large-conductance calcium-activated potassium current (K_Ca_), BK, that is larger than in phasic neurons. Based on these observations, we propose a cellular model in which expression of a fast BK current plays a critical role in shaping PSPs and suppressing spike frequency adaptation, thereby regulating IPI tuning in tonic neurons (**Figure 10**).

### INTRINSIC PROPERTIES COMBINE WITH SYNAPTIC MECHANISMS TO ESTABLISH INTERVAL TUNING

In the mormyrid ELp, previous studies have largely focused on network connections and synaptic mechanisms that establish IPI tuning (George et al., 2011; Ma et al., 2013). By combining whole-cell recording from brain slice, pharmacology, and computational modeling, George et al. (2011) suggested that temporal summation of excitatory and inhibitory input, and relative timing of the two competing inputs can generate diverse IPI tuning types, e.g., high-pass or low-pass. More recently, Ma et al. (2013) developed a whole-brain preparation in which local network connections in ELp remain intact. They performed dual whole-cell recording from synaptically connected ELp neurons and found extensive local excitatory network interactions that influence IPI tuning.

In the present study, we focused on how synaptic inputs that result from network interactions are integrated and converted into action potential output within individual ELp neurons. Our results demonstrate that differences in intrinsic excitability relate to the shape of IPI tuning curves (sharpness of PSP tuning and PSP-to-spike conversion; **Figures 1–4**), rather than IPI tuning type. Thus, an emerging model is that synaptic and network mechanisms grossly determine IPI tuning type, with active postsynaptic membrane properties fine-tuning this temporal selectivity. Effects of Ni^2+^ application on spike frequency adaptation, action potential waveform, and voltage-gated currents suggest that both tonic and phasic neurons express voltage-gated Ca (Ca_v_) and K_Ca_ channels, but tonic neurons express a larger fast-transient K_Ca_ current than phasic neurons (**Figures 5–7** and **10A**, top). Responses to application of a selective blocker, paxilline, strongly suggest that a fast BK current is an essential component of the K_Ca_ currents that establish tonic firing patterns (**Figures 8** and **9**).

Based on these observations, we propose a cellular model to explain how the variation in BK current contributes to temporal filtering in ELp (**Figure 10B**). During synaptically evoked depolarization below spike threshold (**Figure 10B**, left, see also **Figure 3**), Ca^2+^ influx through Ca_v_ channels activates the BK channels. The open BK channels increase membrane conductance (g_BK_) and thus attenuate the voltage change elicited by synaptic current. This effect occurs in a depolarization-dependent manner owing to the voltage dependency of Ca_v_ channels and BK channels themselves, meaning that this effect is strongest at the best IPI and weakest at the worst IPI. This dampening effect strongly impacts synaptic responses in tonic neurons due to larger BK expression, resulting in a flattened IPI tuning curve.

Once tonic neurons fire an action potential, the fast BK current rapidly repolarizes the membrane potential and suppresses spike frequency adaptation by reducing Na^+^ channel inactivation. By contrast, BK current in phasic neurons is too small to prevent ongoing spikes from adapting (**Figure 10A**, bottom). Thus, compared to phasic neurons, tonic neurons are more capable of encoding large and/or long-lasting synaptic depolarizations into a large number of spikes, resulting in less discrepancy between spike and synaptic IPI tuning curves (**Figure 2C**). This effect is prominent particularly at short IPIs (**Figure 10B**, right, see also **Figure 2D**) at which synaptic responses readily summate to establish long-lasting depolarizations (George et al., 2011).

Unfortunately, however, we were unable to directly test how blocking BK channels might influence IPI tuning. There are dense excitatory connections among ELp neurons that strongly contribute to IPI tuning (Ma et al., 2013). Thus, bath application of paxilline, which will affect firing patterns throughout the network, would drastically alter the temporal pattern of presynaptic inputs. As a result, if tuning was affected by drug application, we would not be able to conclude that this change is due to blockade of the channels specifically on the recorded neuron. Instead, these changes would reflect changes in the tuning of the recorded neuron as well as presynaptic neurons.

### IONIC CONDUCTANCES SHAPING TEMPORAL PROCESSING

The functional significance of membrane properties in temporal processing has been particularly well described in auditory systems where neurons exhibit selective responses to sound frequency, interaural time difference, and amplitude-modulation (AM) frequency (Trussell, 1999; Kuba, 2007; Johnston et al., 2010; Ponnath and Farris, 2010; Voigt and Zheng, 2010; Ashida and Carr, 2011; Brown and Kaczmarek, 2011; O’Donnell and Nolan, 2011; Golding, 2012). In the electrosensory system of wave-type electric fishes, Ca^2+^-dependent (Ellis et al., 2007; Krahe et al., 2008; Mehaffey et al., 2008) and voltage-dependent conductances (Fortune and Rose, 1997, 2003; Carlson and Kawasaki, 2006; Nogueira and Caputi, 2013) are also suggested to regulate frequency and AM-frequency tuning. To our knowledge, this study is the first to directly examine the intrinsic membrane properties that underlie selectivity for interspike intervals. This was made possible by a unique feature of the ELp preparation in which cellular mechanisms for the processing of precisely controlled, behaviorally relevant inputs can be studied *in vitro*.

To our knowledge, the present study is the first to suggest a role for BK channels in temporal processing of sensory input, at the levels of integrating synaptic inputs into PSPs and of the PSP-to-spike conversion. Among K_Ca_ channel subtypes, the function of small-conductance K_Ca_ (SK) channels has been extensively studied in temporal processing circuits. In the hindbrain neurons of South American wave-type electric fish, SK current opposes burst firing and promotes regular firing, resulting in a selective reduction in low-frequency tuning (Ellis et al., 2007; Mehaffey et al., 2008). In a computational model of cricket auditory neurons, decreasing SK current broadens selectivity for AM frequency toward high-pass, while increasing SK current narrows the selectivity toward low-pass due to enhanced spike frequency adaptation at high-frequency AM (Ponnath and Farris, 2010). However, our results suggest that SK channels are not responsible for the observed differences between tonic and phasic ELp neurons: the difference in Ni^2+^-sensitive outward currents is mostly due to a fast-activating and rapidly inactivating component (**Figure 7**) that is orders of magnitude faster than those of SK currents. Typically, recombinant SK channels show slow activation time constants of 5–15 ms and deactivation time constants of ∼50 ms in response to Ca^2+^ application (reviewed in Xia et al., 1998; Adelman et al., 2012).

Instead, our results suggest that a fast transient BK current contributes to interval tuning by influencing both synaptic and spiking responses. In addition to the sensitivity to paxilline (**Figures 8** and **9**), the fast kinetics of the Ni^2+^-sensitive and paxilline-sensitive outward currents are in line with those of BK currents: the fast-activation time constant of recombinant BK channels is typically ∼1 ms (e.g., Behrens et al., 2000). Kinetics of BK currents can be substantially modified by auxiliary β-subunits, BKβ (reviewed in Torres et al., 2007; Berkefeld et al., 2010). In particular, coexpression of BKβ3 subunits with α-subunits results in rapidly and partially inactivating K_Ca_ currents (time constant: ∼1 ms; Uebele et al., 2000; Xia et al., 2000; Lingle et al., 2001), similar to the fast-decaying phase of the Ni^2+^-sensitive and paxilline-sensitive outward currents that we observed (**Figures 7–9**). Finally, despite the hyperpolarizing effect of BK current, studies over the past decade have suggested a role for BK current in promoting repetitive firing and thus potentially opposing spike frequency adaptation, as we proposed in **Figure 10**. In rodent brain neurons, genetically induced (Brenner et al., 2005) or seizure-induced (Shruti et al., 2008) gain-of-function in BK channels is associated with elevated firing rate. On the other hand, pharmacological block of BK channels reduces firing rate in canine intracardiac neurons (Perez et al., 2013). By combining pharmacology and computational modeling, Gu et al. (2007) further demonstrated that BK currents suppress early spike frequency adaption, particularly at high firing rates. The present study is the first to suggest that this novel mechanism of BK-channel control over spike frequency adaptation plays a significant role in behaviorally relevant network function.

One intriguing question is the subcellular distribution of channels that enables modification of interval tuning at two different levels of processing, synaptic integration and PSP-to-spike conversion. Our pharmacological results regarding firing pattern and spike shape suggest that the BK currents we recorded were mostly perisomatic. Perisomatic BK channels also explain the observed effects on synaptic responses (**Figure 10B**, left), but it is also possible that dendritic BK channels (Johnston and Narayanan, 2008) shape local dendritic computations and influence the integrated somatic synaptic response. In this scenario, there may be no direct causal relationship between perisomatic BK expression, i.e., spike frequency adaptation, and the sharpness of PSP tuning curves. Separating dendritic or somatic BK currents by focal application of channel-specific blockers could answer these questions.

Our voltage-clamp experiments also suggest that phasic neurons express a low-voltage-gated K_v_ current that is larger than in tonic neurons (**Figure 7**, +TTX+Ni^2+^). K_v_ currents also control spike frequency adaptation (reviewed in Rudy and McBain, 2001; Baranauskas, 2007). In particular, over-expression of the low-voltage-gated Kv1 α-subunit promotes phasic firing in rodent neurons (Malin and Nerbonne, 2001), directly demonstrating that high expression of particular Kv currents can contribute to phasic firing. Functional roles of K_v_ current in temporal processing have been well described in auditory brainstem, including many computational processes other than spike frequency adaptation such as controlling spike timing, shaping postsynaptic responses and setting resting membrane properties (reviewed in Johnston et al., 2010; Voigt and Zheng, 2010; Golding, 2012). Overall, we suggest that balanced expression of K_Ca_ currents, particularly BK currents, which promote tonic firing and provide calcium-dependent PSP regulation, and K_v_ currents, which facilitate phasic firing and provide voltage-dependent PSP regulation, establish the observed range of spike frequency adaptation and IPI tuning among ELp neurons.

## AUTHOR CONTRIBUTIONS

Tsunehiko Kohashi performed experiments and analyses. Tsunehiko Kohashi and Bruce A. Carlson contributed to conceptualization and discussion of the experiments and analyses. The manuscript was written by Tsunehiko Kohashi and Bruce A. Carlson.

## Conflict of Interest Statement

The authors declare that the research was conducted in the absence of any commercial or financial relationships that could be construed as a potential conflict of interest.
